# A Cross-Sectional Study Conducted in the USA Examining Associations Between Religious Engagement and Inpatient Hospital Experience and 30-Day Readmission

**DOI:** 10.1007/s10943-025-02429-w

**Published:** 2025-08-27

**Authors:** Brook E. Harmon, Jonathan Lewis, Jamaica Ivery-Glover, Briana Baptist, Rachel Daniel, Jenna Warnock, Stacy Smith, Sherrhoda Townsend, Sandra Madubuonwu

**Affiliations:** 1 Department of Nutrition and Health Care Management, Appalachian State University, Leon Levine Hall, Room 575, 1179 State Farm Rd, Boone, NC 28607 USA; 2 Methodist Le Bonheur Healthcare, Memphis, TN USA; 3 Porter-Leath, Memphis, TN USA; 4 Department of Human Nutrition, Foods, and Exercise, Virginia Polytechnic Institute and State University, Blacksburg, VA USA; 5 Presbyterian Church (USA), New Haven, CT USA

**Keywords:** Religiosity, Spirituality, African Americans, Hospital, Spiritual care

## Abstract

Associations between the Duke University Religion Index (DUREL) and patients’ perceptions of care, requesting to see a chaplain, and 30-day readmissions were examined in this cross-sectional study. Participants were recruited from an inpatient setting in Memphis, TN and asked the DUREL, three patient experience questions, and if they would like to see a chaplain. The electronic medical record was monitored for readmissions within 30 days of discharge. Logistic regression models included age, length of stay, sex, race, insurance status, and rating of overall health. Two models examining requests to see a chaplain were run. One model included if a chaplain had previously been seen. Associations with DUREL subscale scores (attendance, non-organized activities, intrinsic religiosity) were examined if associations with the total score were statistically significant. Participants (*n* = 482) were on average 62 years old and most identified as female and Black. Higher total DUREL scores were associated with greater confidence in enacting care plans, with attendance and intrinsic religiosity scores driving the association. Participants with higher total DUREL scores and longer hospital stays were more likely to request a chaplain. Higher attendance and non-organized activity subscale scores were associated with making a request. No statistically significant association was identified for 30-day readmission. Findings add to our understanding of the role religious engagement plays within inpatient settings and to the literature emphasizing the role of religious and spiritual discussions during discharge. More research is needed to see if confidence translates into better health post-hospital stay.

## Introduction

Religiosity refers to the intensity of religious beliefs, practices, and rituals related to a higher power that is held by an individual or a community (Koenig et al., 2012). It is often examined by measuring levels of devotion, observance, and adherence to established religious traditions [e.g., Christianity, Islam, Hinduism, Judaism] (Koenig et al., 2012; Masters & Hooker, 2013). Definitions of spirituality have been inconsistent in the literature; however, it has traditionally been defined as the inward or personal pursuit of faith or the personal or collective pursuit of the sacred (Koenig, 2013; Malinakova et al., 2020; Zinnbauer & Pargament, 2005). Religious engagement [e.g., public religious attendance, membership, participation] has been the most frequently used measure of religiosity and spirituality (R/S) when examining religiosity and health (Masters & Hooker, 2013). A high level of religious engagement has been found to be associated with reduced mortality and a variety of other positive mental and physical health outcomes (Koenig, 2013; Masters & Hooker, 2013; McCullough et al., 2000).

Much of the research within inpatient settings has focused on R/S and patient experience (Gad et al., 2020; Hodge & Wolosin, 2015; Hodge et al., 2016; Williams et al., 2011), with patient experience serving as a proxy for service quality (Batbaatar et al., 2016). Addressing the R/S needs of patients has been found to be associated with higher patient satisfaction in several studies (Hodge & Wolosin, 2015; Hodge et al., 2016; Williams et al., 2011). However, research to-date has primarily been conducted in the early and mid-2000s, and since then, the USA has seen a growth in individuals who identify as religiously unaffiliated (Pew Research Center, 2024). Despite this shift, research indicates individuals with no religious identity still desire conversations about their spiritual care (Gad et al., 2020; Muehlhausen et al., 2021). These findings highlight the complicated and little understood relationships that exist between patients’ religious identities, expectations of care, and health outcomes in healthcare settings (Batbaatar et al., 2016; Masters & Hooker, 2013). To aid research in this area, the integration of religious screeners in electronic medical records (EMR) has become an area of interest (Puchalski, 2001; Stewart et al., 2022; Tartaglia et al., 2016; Woggon et al., 2022), but this work has been complicated by the need for training, time, integration into current business models, resources, and privacy concerns (Hawthorne & Gordon, 2020; Puchalski et al., 2019; Saguil & Phelps, 2012).

In this study, the Duke University Religion Index (DUREL), a validated (Toscanelli et al., 2022) and extensively used (Kopacz et al., 2017; Young & Clark, 2013) religious engagement measure, was incorporated into the EMR of a healthcare system. The relationship between religious engagement, as measured by the DUREL, and healthcare-centered outcomes [i.e., perception of overall care, system trust, confidence in enacting care plans, requesting to see a chaplain, 30-day readmissions] were examined.

## Methods

Participants were recruited from two hospitals in a 1687-bed healthcare system in Memphis, TN. These two hospitals were selected due to their support of past programs and status in the community as anchor providers. Data collection, began at Hospital A, was collected for 2 months, and then a second coordinator was hired to oversee data collection at Hospital B. Data were collected between December 2023 and March 2024. Using each location’s daily census, the outreach coordinators checked inpatients against the EMR to determine eligibility for the study. Eligible participants were 18 years or older, able to read and understand English, without cognitive impairment due to a recent stroke or dementia, and not in the emergency room or under observation at the time of approach. Ineligible individuals included those who were COVID-19 positive, in critical care units, had a medical device hindering communication (e.g., ventilator), or participated during a previous stay. Internal Review Board (IRB) approval was provided by the University of Tennessee Health Sciences Center (IRB# 22–08651-XP), the healthcare system’s IRB of record.

Using a cross-sectional survey, eligible patients were asked a series of questions that included overall health perception, patient experience, how participants cope with pain, and two open-ended questions about spirituality practices. Data on demographics, length of stay, and 30-day readmission were collected from the EMR. To measure religious engagement, the 5-item DUREL was asked (Koenig & Büssing, 2010). The DUREL is designed to measure three dimensions of religious engagement: organizational religious activity (ORA), non-organizational religious practices (NORA), and intrinsic religiosity (IR) (Koenig & Büssing, 2010). Evaluation of the three dimensions results in three subscale scores as well as an overall score that ranges from 5 to 27, where higher scores suggest a higher level of religious engagement (Koenig & Büssing, 2010).

To assess patient experience, three questions were adapted from the *Hospital Consumer Assessment of Healthcare Providers and Systems (*HCAHPS: Patients’ Perspectives of Care Survey, 2023*)*. The questions assessed perception of overall care [i.e., “During your stay, how would you rate your overall care?”], system trust [i.e., “I trust the Methodist Le Bonheur Healthcare system.”], and confidence in implementing care plans post-stay [i.e., “I am confident in my plans for care after I leave the hospital.”]. At the end of the interview, participants were asked if they would like to speak with a chaplain. The EMR was checked at 31 days after discharge to see if participants were readmitted within 30 days of their participation in the study. If a participant was readmitted, this was recorded followed by the reason for readmittance.

The outcomes requesting a chaplain and 30-day readmission were naturally dichotomous outcomes (yes/no). Given the distribution of the patient experience data, these outcomes were also dichotomized. For overall experience, responses of Poor/Fair/Uncertain were categorized as low and Good/Excellent as high. For trust and confidence in care plans, Neutral/Disagree/Strongly Disagree were categorized as low and Strongly Agree/Agree were categorized as high. Logistic regressions using SPSS version 29 were used to examine the study’s aims.

The first models examined associations between the DUREL total score and the three hospital experience variables (overall care, system trust, confidence in care plans). Models included age, length of stay, sex (male, female), race (black, other), insurance status (yes, no), and rating of overall health (good–excellent, fair/poor). This same model was used to examine the DUREL total score and 30-day readmission. The model examining the DUREL total score and participants’ requesting to see a chaplain included the same variables except insurance status was removed. As some patients had already seen a chaplain before participating in the study, two versions of the model examining requesting to see a chaplain were run. Model 1 did not include the variable assessing if patients had already seen a chaplain (yes, no) and model 2 included the variable. A previous visit from the chaplain was assessed by asking participants as well as checking the EMR for a spiritual care note that preceded the study visit. If the total DUREL score was significantly associated with the outcome of interest, models were run with each of the three subscales (attendance, non-organized activities, intrinsic religiosity) to see which aspects of the DUREL score were driving associations. Missing data were excluded from analyses. Statistical significance was set at p ≤ 0.01 due to the number of associations being examined in this study.

## Results

Over the course of the study, 1264 patients were approached, 902 were eligible, and 482 participated (53.4% response rate). Most participants who declined to participate did not give a reason for declining. The average age of participants was 61.7 years (SD = 14.4) and most identified as African American (71%), female (59.5%), and had insurance (89.6%). The average hospital stay was 8 days long (SD = 6.4) and most participants were not readmitted within 30 days (83.1%) although they rated their health during their stay as fair or poor (61.4%). See Table 1 for characteristics of the sample.

**Table 1:** Demographics of participants enrolled in the study (*n* = 482)

Characteristics	Mean (SD) or N (%)^b^
Age	61.7 (14.4)
DUREL^a^ Score	21.6 (4.1)
Length of Stay	8 days (6.4)
Sex	
Female	287 (59.5%)
Male	193 (40%)
Race/Ethnicity	
African American	342 (71%)
Other^c^	137 (28.4%)
Overall Health Rating	
Good to excellent	184 (38.2%)
Fair/Poor	296 (61.4%)
30-Day Readmission	
Yes	81 (16.8%)
No	401 (83.1%)
Insurance Status	
Yes	432 (89.6%)
No	50 (10.4%)
Requested a Chaplain	
Yes	116 (26%)
No	364 (75.5%)
Already Seeing a Chaplain	
Yes	67 (14%)
No	350 (73%)
Perception of Overall Care^d^	
High perception of care	392 (81.3%)
Low perception of care	75 (15.6%)
System Trust^e^	
High Trust	408 (84.6%)
Low Trust	59 (12.2%)
Confidence in Care Plans^e^	
High Confidence	286 (59.3%)
Low Confidence	177 (36.7%)

^a^Duke University Religion Index. ^b^Percentages may not equal 100 due to missing data. ^c^Other included individuals who selected Other as well as American Indian/Alaskan Native, Asian, Hispanic, Native Hawaiian/Pacific Islander, and White. ^d^Responses of Poor/Fair/Uncertain were categorized as low and Good/Excellent as high. ^e^Responses of Neutral/Disagree/Strongly Disagree were categorized as low and Strongly Agree/Agree were categorized as high

On average, DUREL scores were high (21.6 (SD = 4.1), and most participants rated their overall care as high (81.3%) as well as their trust in the system (84.6%). While over half, a lower percentage of participants had high confidence in their care plans (59.3%). In the models examining patient experience (see Table 2), DUREL total scores only had a statistically significant association with confidence in enacting care plans. Individuals with higher DUREL scores had higher odds of being confident in enacting their care plans compared to individuals with lower DUREL scores (OR = 1.09, 95% CI = 1.04–1.15). When the DUREL subscales were examined (see Table 2), attendance (OR = 1.19, 95% CI = 1.06–1.34) was positively associated with confidence in enacting care plans as was IR (OR = 1.47, 95% CI = 1.11–1.96,). No other variables in the models were significantly associated with patient experience outcomes.

**Table 2:** Findings from logistic regressions examining religious engagement and healthcare-centered outcomes^a^

Covariates	30-day readmission	Overall care	System trust	Confidence in care plans	Requesting a chaplain (model 1)^b^	Requesting chaplain (model 2)^b^
**DUREL**^**c**^ **Total Score**	1.02 (0.95,1.09)	1.03 (0.97,1.10)	1.03 (0.96,1.10)	1.10 (1.04,1.15)^*^	1.11 (1.03, 1.18)^*^	1.12 (1.04,1.21)^*^
Age	1.01 (0.99, 1.03)	1.00 (0.98, 1.02)	1.00 (0.98, 1.02)	1.00 (0.98, 1.01)	1.00 (0.98, 1.02)	1.00 (0.98, 1.02)
Sex	1.01 (0.60, 1.71)	1.08 (0.63, 1.86)	0.77 (0.42, 1.41)	1.04 (0.69, 1.58)	1.51 (0.94, 2.43)	1.91 (1.12, 3.25)^*^
Race	1.10 (0.62, 1.93)	0.57 (0.31, 1.07)	0.85 (0.44, 1.62)	1.19 (0.77, 1.84)	1.44 (0.85, 2.44)	1.55 (0.85, 2.82)
Length of stay	1.03 (1.00, 1.07)	0.98 (0.94, 1.01)	0.98 (0.94, 1.02)	0.98 (0.95, 1.01)	1.05 (1.01, 1.08)^*^	1.07 (1.03, 1.11)^*^
Overall health	0.86 (0.51, 1.43)	1.57 (0.91, 2.71)	2.16 (1.14, 4.10)	1.39 (0.93, 2.07)	0.75 (0.47, 1.19)	0.68 (0.41, 1.13)
Insurance status	0.37 (0.11, 1.26)	0.91 (0.38, 2.16)	1.21 (0.43, 3.38)	0.94 (0.48, 1.84)	–	–
Seen a chaplain	–	–	–	–	–	0.05 (0.01, 0.20)^*^
Subscales						
**Attendance**				1.18 (1.05,1.33)^*^	1.19 (1.04, 1.36)^*^	1.28 (1.10,1.49)^*^
Age				1.00 (0.98, 1.01)	1.00 (0.98, 1.02)	1.00 (0.98, 1.02)
Sex				1.16 (0.78, 1.74)	1.65 (1.03, 2.64)	2.14 (1.27, 3.63)^*^
Race				1.20 (0.78, 1.85)	1.46 (0.86, 2.47)	1.53 (0.84, 2.79)
Length of stay				0.98 (0.95, 1.01)	1.04 (1.01, 1.08)^*^	1.07 (1.02, 1.11)^*^
Overall health				1.38 (0.92, 2.06)	0.75 (0.48, 1.20)	0.69 (0.41, 1.14)
Insurance status				1.01 (0.52, 1.98)	–	–
Seen a chaplain				–	–	0.05 (0.01, 0.21)^*^
**NORA**^**d**^				1.11 (0.99,1.26)	1.22 (1.05, 1.43)^*^	1.21 (1.02,1.45)
Age				1.00 (0.99, 1.02)	1.00 (0.99, 1.02)	1.00 (0.99, 1.02)
Sex				1.15 (0.77, 1.73)	1.58 (0.99, 2.53)	2.02 (1.19, 3.43)^*^
Race				1.33 (0.87, 2.04)	1.61 (0.96, 2.71)	1.82 (1.02, 3.27)
Length of stay				0.98 (0.95, 1.01)	1.05 (1.02, 1.08)^*^	1.07 (1.03, 1.12)^*^
Overall health				1.41 (0.95, 2.10)	0.77 (0.48, 1.21)	0.70 (0.42, 1.15)
Insurance status				0.91 (0.46, 1.77)	–	–
Seen a chaplain				–	–	0.05 (0.01, 0.21)^*^
**IR**^**e**^				1.49 (1.13–1.97)^*^	1.31 (0.91, 1.89)	1.36 (0.91–2.03)
Age				1.00 (0.99, 1.02)	1.00 (0.99, 1.02)	1.00 (0.99, 1.02)
Sex				1.11 (0.73, 1.67)	1.66 (1.04, 2.66)	2.13 (1.26, 3.59)^*^
Race				1.28 (0.83, 1.96)	1.61 (0.95, 2.70)	1.77 (0.98, 3.19)
Length of stay				0.98 (0.95, 1.01)	1.05 (1.01, 1.08)^*^	1.07 (1.03, 1.11)^*^
Overall health				1.43 (0.96, 2.13)	0.79 (0.50, 1.24)	0.72 (0.44, 1.18)
Insurance status				0.95 (0.48, 1.86)	–	–
Seen a chaplain				–	–	0.05 (0.01, 0.21)^*^

^a^All models included: age, sex (male, female—predictor), race (Black—predictor, other), length of stay, rating of overall health (good–excellent—predictor, fair/poor), insurance status (yes, no-predictor). ^b^Requesting a chaplain model 1 did not include insurance status, Requesting a chaplain model 2 did not include insurance status and did include if participants had previously seen a chaplain (yes—predictor, no). ^c^Duke University Religion Index. ^d^NORA = non-organizational religious practices. ^e^IR = intrinsic religiosity. ^*^p ≤ 0.01

In both models, participants with higher DUREL total scores were more likely to request a chaplain (Model 1: OR = 1.11, 95% CI = 1.03–1.18; Model 2: OR = 1.12, 95% CI = 1.04–1.21) than individuals with lower scores (see Table 2). Participants with longer stays were also more likely to request a chaplain (Model 1: OR = 1.05, 95% CI = 1.01–1.08; Model 2: OR = 1.07, 95% CI = 1.03–1.11). In Model 2, participants who were female were more likely to request a chaplain (OR = 1.91, 95% CI = 1.12–3.25) while participants who had already seen a chaplain were less likely to place a request (OR = 0.05, 95% CI = 0.01–0.20). Sex was not significant in model 1 (OR = 1.51, 95% CI = 0.94, 2.43).

When examining the DUREL subscales, attendance was significantly associated with requesting a chaplain in both models. Compared to individuals with lower religious attendance scores, individuals with higher scores were more likely to request a chaplain (Model 1: OR = 1.19, 95% CI = 1.04–1.36; Model 2: OR = 1.28, 95% CI = 1.10–1.49). In model 1, the NORA subscale was also significant such that individuals with higher participation in activities “such as prayer, meditation, and Bible study” were more likely to request a chaplain (Model 1: OR = 1.22, 95% CI = 1.05, 1.43). A similar association was seen in model 2; however, it was not statistically significant at *p* ≤ 0.01 (Model 2: OR = 1.21, 95% CI = 1.02, 1.45, *p* = .03). Covariates remained statistically significant across the subscale models (Model 1: length of stay; Model 2: sex, length of stay, previously seeing a chaplain) with the same direction of association as seen in the DUREL total score models (see Table 2).

No statistically significant difference was observed between the DUREL and readmission within 30 days of the current stay (see Table 2).

## Discussion

This study examined associations between the DUREL and patient hospital experience, requesting to see a chaplain, and 30-day readmission. Higher DUREL scores were associated with higher confidence in enacting care plans and requesting a chaplain, but DUREL scores were not associated with the other patient experience outcomes or 30-day readmission. These findings add to our understanding of how religious engagement contributes to healthcare-centered outcomes.

While religious engagement was not associated with all measures of patient experience in this study, DUREL scores were positively associated with patients’ confidence in their ability to enact their care plans. Previous research with samples of older adults and African Americans has found discharge to be a part of the inpatient experience where patients expect their spiritual needs to be met (Hodge & Wolosin, 2015; Hodge et al., 2016). Among older adults, patients’ spiritual needs were directly and positively associated with higher levels of overall satisfaction with their hospital experience, and the association was mediated by a variety of services, including discharge (Hodge et al., 2016). In another study, discharge was one area of inpatient service that predicted dissatisfaction in having spiritual needs met among African American patients (Hodge & Wolosin, 2015). Previous research has also found African American patients may be less likely to have spiritual concerns discussed with them during their stay (Williams et al., 2011). Given our findings, future research should examine interventions that increase R/S discussions during discharge and care planning.

The association between religious engagement and confidence in enacting care plans was driven by higher attendance and intrinsic religiosity scores. Previous research has found individuals with higher intrinsic religiosity are able to place their individual experiences into a broader social perspective, which manifests as greater self-confidence, hope, and personal control (Unantenne et al., 2013). These general feelings of optimism and control may play a role in patients feeling confident in managing their care once they leave the hospital. While intrinsic religiosity has been shown to mediate the association between religious attendance and health outcomes such as well-being (Steffen et al., 2017), social support is another construct which may help explain the health benefits of religious attendance. Several studies have found, especially among African Americans, being active in a congregation can be a source of several types of social support [i.e., instrumental, emotional, spiritual] (Krause, 2008; Van Olphen et al., 2003). Future research should examine the pathways through which intrinsic religiosity and religious attendance are impacting patients’ beliefs related to their care plans and ability to enact their care plans at home.

In the two models that examined requesting to see a chaplain, patients with higher DUREL scores were more likely to make a request. This was especially true for individuals with high attendance subscale scores and longer stays. Individuals who regularly attend services may be more comfortable seeking out spiritual care from a faith leader such as a chaplain (Henderson et al., 2023). In addition, patients often reach out to hospital chaplains after receiving a critical diagnosis or undergoing intensive care (Muehlhausen et al., 2021); events that can result in longer hospital stays. When previously seeing a chaplain was included in the models, the association between engaging in more non-organized religious activities [e.g., prayer, meditation, Bible study] and requesting a chaplain was no longer statistically significant while females were significantly more likely to request a chaplain than males. Seeking someone to pray with or for them is often a reason patients give when requesting a chaplain (Henderson et al., 2023; Silton et al., 2013), and research indicates females are more likely to seek out spiritual support than males (Sprik et al., 2018). Additional studies are needed to examine the DUREL’s NORA subscale and sex as predictors of requesting a chaplain during a hospital stay. This is particularly important as previous research indicates chaplain visits are associated with increased patient satisfaction; however, patients are not always sure how to contact a chaplain (Muehlhausen et al., 2021). Research identifying best practices for communicating about chaplain services in inpatient settings is needed.

### Limitations

There are several limitations to consider when examining the study’s findings. Data were cross-sectional, which limits the inferences that can be made. Participants reported high patient satisfaction with overall care and trust and there was a low prevalence of 30-day readmissions, which likely made it difficult to find associations with these outcomes. Patient experience questions in this study were asked during the patient’s stay, which may have impacted responses; however, studies asking similar questions after discharge found similarly high patient experience ratings (Hodge et al., 2016). Questions were asked by community outreach coordinators and not members of the healthcare team, but religious individuals may respond in socially desirable ways about engagement on interview administered questionnaires (Steffen et al., 2017). Our sample was primarily older adults, African Americans, and females, and data were collected from one healthcare organization, which limits the generalizability of the findings to other demographic groups.

## Conclusions

Despite these limitations, the study examined associations using the valid, reliable, and commonly used DUREL. In addition, religious engagement and associations with healthcare-related outcomes were examined, which adds to our understanding of the role of spiritual care within inpatient settings. Higher DUREL scores were associated with increased confidence in enacting care plans and with requesting a chaplain during the hospital stay. The DUREL subscale attendance was positively associated with both outcomes, and intrinsic religiosity was positively associated with confidence in enacting care plans. More research is needed to see if confidence translates into better health post-hospital stay. In addition, future research should explore whether R/S discussions during discharge enhance patient outcomes, and the roles spiritual care teams could play in this process.
